# The Importance of Preserving the Posterior Ligament Complex in Elective Lumbar Fusion Surgery: Early Results from a Single-Center Experience

**DOI:** 10.7759/cureus.76252

**Published:** 2024-12-23

**Authors:** Periklis Godolias, Jonathan Plümer, Charlotte Cibura, Julius Gerstmeyer, Hansjörg Heep, Marcel Dudda, Clifford Pierre, Thomas A Schildhauer, Rod J Oskouian, Jens Chapman

**Affiliations:** 1 Department of Orthopedics and Trauma Surgery, St. Josef Hospital Essen-Werden, Essen, DEU; 2 Department of Orthopedics and Trauma Surgery, BG University Hospital Bergmannsheil, Ruhr University Bochum, Bochum, DEU; 3 Department of Trauma, Hand, and Reconstructive Surgery, University Hospital Essen, Essen, DEU; 4 Department of Trauma and Orthopedic Surgery, University Hospital Essen, Essen, DEU; 5 Department of Neurosurgery, Swedish Neuroscience Institute, Seattle, USA; 6 Department of Orthopedics and Trauma Surgery, BG University Hospital Bergmannsheil, Bochum, DEU

**Keywords:** adjacent segment disease, decompression, laminectomy, lumbar fusion, posterior ligamentous complex

## Abstract

Background: Adjacent segment disease (ASD) is a degenerative condition at the segment adjacent to a previously fused segment. Potential risk factors for ASD, such as posterior ligamentous complex (PLC) integrity between the upper instrumented vertebra (UIV) and the first unfused segment (UIV+1), have not been addressed. The objective of this study is to assess the PLC integrity between the UIV and UIV+1 following posterior lumbar decompression and fusion (PLDF).

Methods: A retrospective review of 122 patients who received a PLDF was performed. Patients were divided into groups based on the integrity of the PLC between the UIV and the UIV+1: PLC disrupted and PLC intact. The development of ASD was assessed using standard radiographic parameters, and reoperation rates were reviewed.

Results: Radiographic indicators for ASD were more common in patients of the PLC-deficient group-D and showed significantly higher mobility at the UIV+1 (p < 0.05). The overall surgical revision rate due to ASD was 7.4%, with group D (28 patients) exceeding the revision rate of group I (94 patients) by 4.3% (10.7% vs. 6.4%) over a mean follow-up of three years. The mean return to the operative report time at the UIV+1 was 2.4 years (± 1.7 years) after index surgery.

Conclusion: We demonstrated a significant increase in mobility at the UIV+1 in lumbar fusion in patients with disrupted PLC. PLC deficiency at UIV+1 appears to contribute to the development of ASD through instability and is implicated in higher surgical revision rates.

## Introduction

Adjacent segment disease (ASD) occurs at the motion unit next to a previously fused segment due to various factors [1,2]. The clinical symptoms include back pain, radicular pain, and radiographic changes of segmental instability [3]. ASD may be symptomatic (sASD) or an asymptomatic variant based on clinical symptoms. Common ASD-related imaging anomalies may present as spondylotic changes such as disc degeneration and herniation, facet joint hypertrophy, segmental instability, and/or deformity with neural compromise [1]. There are knowledge gaps on the etiology of ASD following posterior lumbar decompression and fusion (PLDF) [1]. Pertinent risk factors for ASD include age above 60 years, obesity, poor postural trunk control, and spine-specific pathologies such as facet joint anomalies, multilevel fusion, ending a spine construct at L5 below a multilevel fusion, the presence of a destabilizing laminectomy adjacent to the fusion, and malalignment of the fused segment. Despite the myriad of risk factors, a more concrete association for ASD has yet to be established [2].

Conspicuously, motion segment integrity between the upper instrumented vertebra (UIV) and the segment above the UIV (first unfused UIV, UIV+1) or disruption from a laminectomy that sacrifices the posterior ligamentous complex (PLC) adjacent to a fusion is a suspected risk factor for ASD [3-5]. The aim of our study is to the specific impact of PLC sacrifice in a lumbar decompression surgery performed at the same time adjacent to the upper level of elective fusion surgery. We hypothesized that preservation of the PLC adjacent to a contemporaneously performed elective lumbar fusion surgery decreased abnormal motion and decreased the likelihood of developing radiographically and clinically relevant ASD.

## Materials and methods

After approval by the institutional review board and a waiver of consent were obtained, we performed a single-center retrospective chart review of 194 consecutive patients 18 years and older who were treated with elective PLDF between January 2015 and December 2016. All procedures were performed by three senior spine surgeons. Far lateral, anterior, and minimally invasive procedures, tumor, trauma, and de novo spinal infection cases were excluded. Of note, all procedures were performed at a single center. The authors of this study are connected through a collaborative multidisciplinary spine research fellowship that brings together experts from various institutions worldwide.

Groups

**Figure 1:**
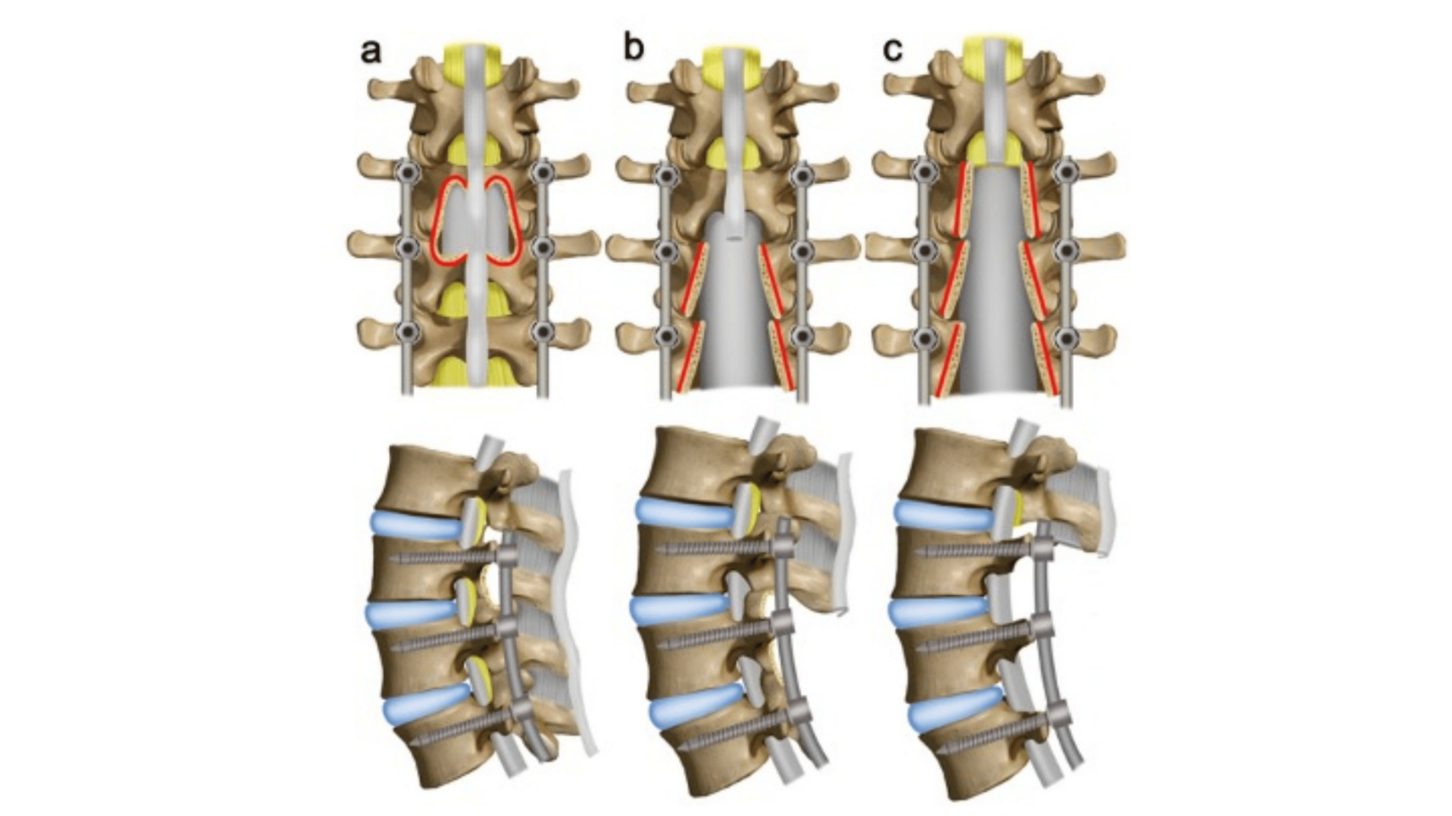
Diagram of laminotomy and fusion. The top panel depicts the posterior view. The bottom panel depicts a sagittal view. (a) Midline sparing laminotomy and fusion of the UIV, PLC intact. (b) PLC intact at the UIV+1. (c) PLC disrupted at the UIV+1 UIV: upper instrumented vertebrae; PLC: posterior ligamentous complex; UIV+1: first unfused segment rostral to the upper instrumented vertebrae Source: Image: © 2024, Seattle Science Foundation. Used with permission from the institution solely for this manuscript

Patients were divided into two study groups based on the integrity of the PLC at the rostral aspect of the construct: PLC disrupted (PLC-D) and PLC intact (PLC-I). PLC-I patients may have had “midline sparing” laminotomies (Figure 1a) of the UIV and/or UIV+1, or the laminae remained intact at the UIV (Figure 1b). PLC-D was defined by resection of the spinous process in conjunction with the removal of the PLC attached to the UIV (Figure 1c). Revision surgeries due to sASD were defined by neurologic symptoms (e.g., worsening lower back pain, new or recurrent persistent radicular pain, and progressive weakness) [6]. Patient assignment to the two subgroups was carried out by evaluation of postoperative computed tomography (CT) scans, plain radiographs, and review of operative reports. Patient characteristics and comorbidities were obtained from the electronic medical record. We also scrutinized the medical records, including outside records for revision surgeries, and formally assessed pre- and postoperative radiographs, including flexion-distraction images. Figure 1 shows the diagram of laminotomy and fusion.

Measurements

**Figure 2:**
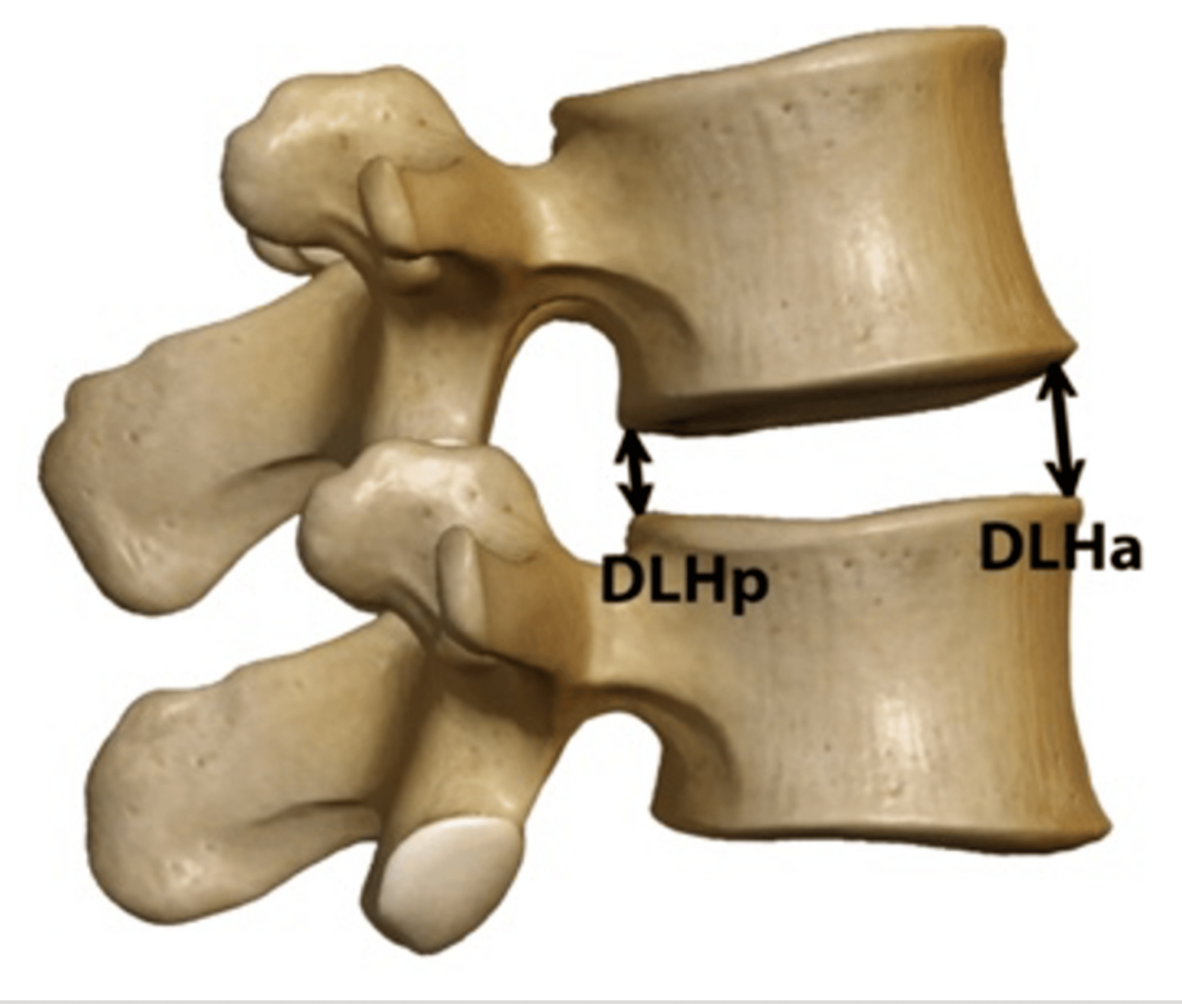
Diagram of measurement of the disc space The formula DH = (DLHa + DLHp) / 2 calculates the average DH between two adjacent vertebrae. DLHa: This represents the distance of the anterior height of the disc, measured from the front (anterior) side of the vertebrae. DLHp: This represents the distance of the posterior height of the disc, measured from the back (posterior) side of the vertebrae DH: disc height; DLHa: disc height anterior; DLHp: disc height posterior Source: Image: © 2024, Seattle Science Foundation. Used with permission from institution solely for this manuscript

All X-ray images were scaled in the Paxera viewer (M-view, Seoul, Korea) using the implant sizes known from the operative reports. Of note is that the preoperative disc height (DH) was measured using CT scans only. CT images used for verification were available in known scaling (pixel/voxel). The changes in DH after the first surgery and at follow-up could be measured using scalable radiograph images. Each radiograph was scaled according to the inserted implants, whose dimensions were known. All measurements were independently performed by a board-certified orthopedic and trauma surgeon who was blinded to surgeons and eventual outcomes. The height of the disc space above the UIV was calculated from a lateral standing X-ray, as shown in the diagram in Figure 2.

**Figure 3:**
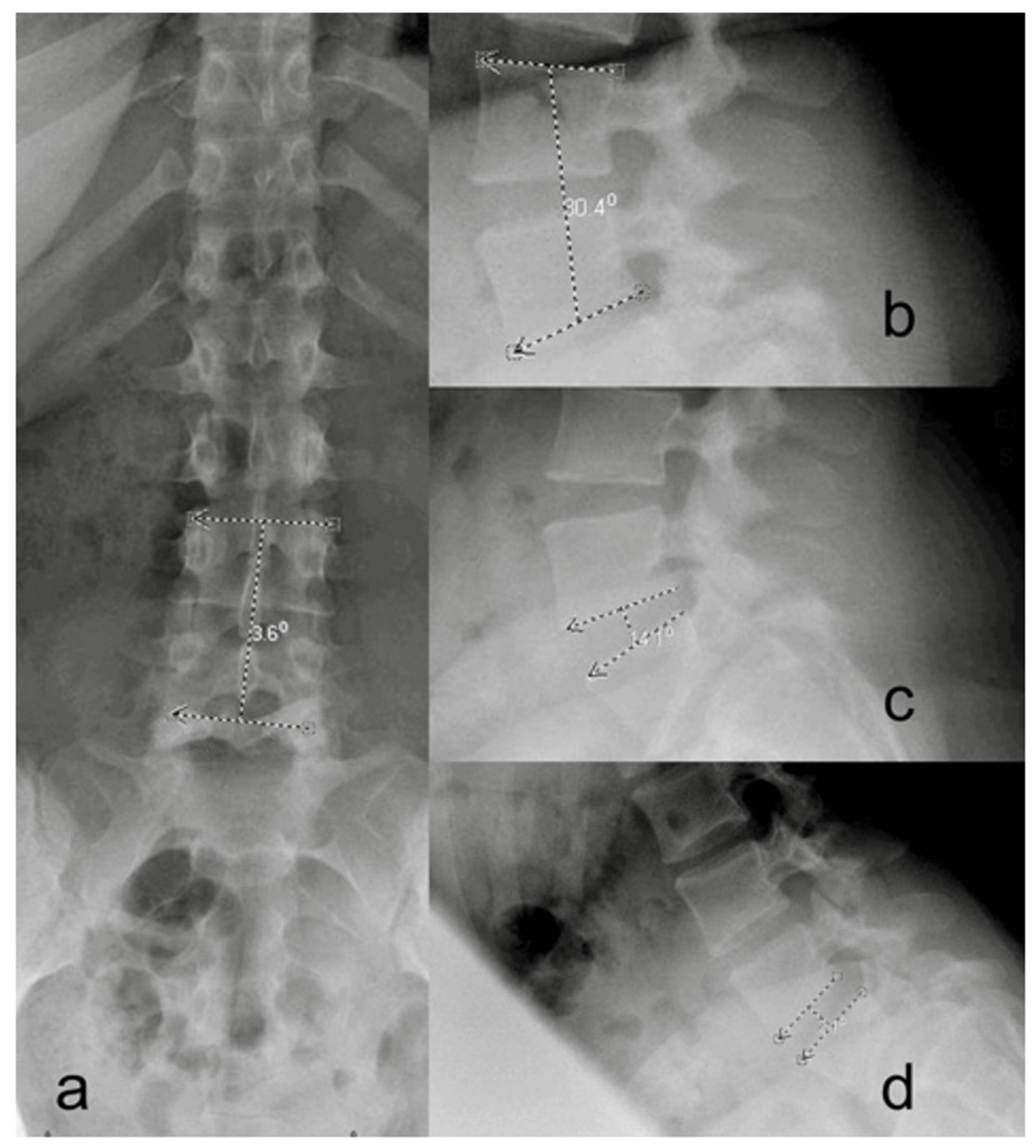
Pre-operative measurements on lateral standing X-ray. (a) Anterior-posterior view. (b-d) lateral view Source: Image: © 2024, Seattle Science Foundation. Used with permission from the institution solely for this manuscript

**Figure 4:**
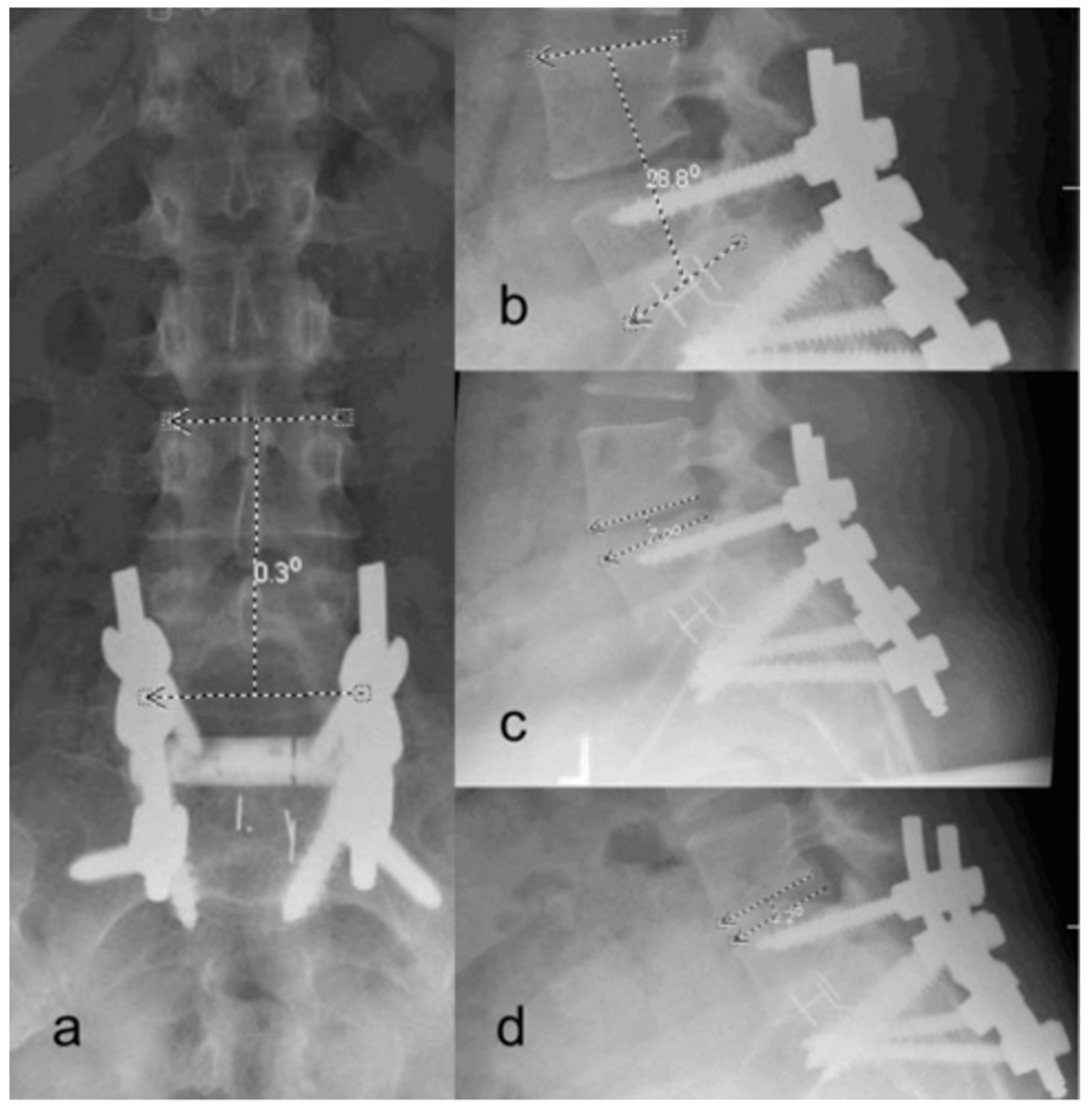
Postoperative measurements on lateral standing X-ray. (a) Anterior-posterior view. (b-d) Lateral view Source: Image: © 2024, Seattle Science Foundation. Used with permission from institution solely for this manuscript

For measurements of the alignment between UIV and UIV+1, anteroposterior, lateral, flexion, and extension, X-rays were used as outlined in Figures 3, 4: segmental wedge angle, segmental lordosis, disc angle at flexion, and disc angle at extension. Further measurements of lateral listhesis of the UIV+1 in the anteroposterior plane and the amount of listhesis in the lateral plane are expressed in absolute and percentage of the vertebral body (VB) width.

Assessment of data between both groups

Patients who received revision surgery due to sASD at the UIV+1 during follow-up were identified, and the following indicators of ASD at the segment between the UIV and the UIV+1 were applied: 1) decrease of DH of ≥20%; 2) increase of VB slippage in the anterior-posterior plane; 3) increase in lateral VB slippage; 4) increase in segmental range of motion (ROM) of ≥20%; and 5) increase in wedge angle [6-8]. The segmental ROM was calculated using the following formula: Delta Sign ROM = [(Disc angle extension - Disc angle flexion) Follow-up / (Disc angle extension - Disc angle flexion) Pre-operative] × 100.

Statistical analysis

Descriptive statistics were applied for the assessment of demographic, radiographic, and postsurgical variables. For categorical variables, frequency counts were computed and presented along with their percentages. For continuous variables, means were computed and presented along with their standard deviation. Statistical tests for categorical variables were performed via chi-square and for the continuous t-tests. We estimated the risk ratio (RR) and 95% confidence interval (CI) of ASD and surgery for sASD, comparing the PLC-D to the PLC-I group, and tested the hypothesis that the RR was equal to 1.0 using Fisher’s exact test. A p value of 0.05 was considered statistically significant. Analyses were performed using Stata software, version 9.0 (StataCorp LLC, College Station, TX). A version of this study was previously posted to the medRxiv preprint server on April 5, 2023, and May 30, 2024.

## Results

The study sample comprised 122 individuals (66 females and 56 males) selected from 194 eligible patients. The analysis excluded 72 patients due to inadequate follow-up information. The average duration of follow-up within the study cohort was three years, while a minimum follow-up period of one year was established (PLC-D: 1,066 days; PLC-I: 1,100 days; range: 367-2,255 days). The mean age at index surgery was 63 (20-86) years. Age, sex, body mass index, time to follow-up, and days to revision due to ASD were equal between both groups, as shown in Table 1. The revision rate due to ASD of the entire cohort (n = 122) was 7.4% (nine patients) within the observed time. The revision rate of PLC-D exceeded the revision rate of group I by 4.3 %. Three patients (10.7 %) of PLC-D and six patients (6.4%) of PLC-I needed revision surgery within an average of 2.4 years (±1.7 years) due to ASD of the UIV+1 level (p = 0.442). All revision surgeries were carried out by rostral extension with fusion of the construct and decompression of the affected segment.

**Table 1:** Demographics and indicators for ASD by treatment PLC: posterior ligamentous complex; BMI: body mass index; sASD: symptomatic adjacent segment disease; ASD: adjacent segment disease

Baseline demographics and indicators	PLC disrupted (n = 28)	PLC intact (n = 94)	p value (t-test)
Age (years), mean (±SD)	61.9 (15.1)	63.7 (12.1)	0.520
BMI (kg/m^2^), mean (±SD)	30.4 (5.5)	28.7 (5.7)	0.161
Follow-up (days), mean (±SD)	1,066.4 (520.9)	1,100.3 (541.1)	0.769
Days to revision due to ASD, mean (±SD)	879.2 (498.2)	860.8 (603.2)	0.947
Female, n (%)	15 (53.6)	51 (54.3)	-
Male, n (%)	13 (46.4)	43 (45.7)	-
Tobacco use, n (%)	13 (46.4)	52 (55.3)	0.408
Diabetes, n (%)	3 (10.7)	18 (19.2)	0.299
Connective tissue disease, n (%)	0 (0.0)	6 (6.7)	0.161
Revisions due to sASD, n (%)	3 (10.7)	6 (6.4)	0.442
3+ indicators for ASD (out of 5), n (%)	18 (64.3)	55 (58.5)	0.584
Decrease in disc height ≥20%, n (%)	13 (46.4)	39 (41.5)	0.643
Increase in vertebral body slippage, n (%)	18 (64.3)	48 (51.1)	0.218
Increase in range of motion ≥20%, n (%)	17 (60.1)	52 (55.3)	0.613
Increase in lateral slippage, n (%)	13 (46.4)	41 (43.6)	0.793
Increase in wedge angle, n (%)	24 (85.7)	72 (76.6)	0.301

While tobacco use and diabetes were distributed equally between both groups, rheumatoid arthritis was unequally distributed, with six cases (6.7%) in the PLC-I group compared to no cases in the PLC-D group (p = 0.161). Our data implied that the relative risk of undergoing revision surgery due to ASD with a PLC-I was 0.6 (95% CI, 0.16-2.23).

As to level distribution, the L4/5 level represented the most frequently instrumented segment for both patient cohorts (36% of PLC-D and 38% of PLC-I). All levels fused in this cohort are provided in Table 1. Our evaluation of ASD demonstrated a higher incidence of this occurrence in PLC-D patients. The relevant findings are displayed in Table 2.

**Table 2:** Average measurements by treatment PLC: posterior ligamentous complex

Measurements	PLC disrupted (n = 28), mean (±SD)	PLC intact (n = 94), mean (±SD)	p value (t-test)
Preoperative			
Disc height	8.7 (2.6)	8.1 (2.5)	0.245
Vertebral body slippage	-0.36 (3.4)	0.16 (3.4)	0.771
Segmental lordosis	18.7 (13.0)	15.2 (9.4)	0.114
Disc angle flexion	6.5 (4.4)	5.3 (4.2)	0.197
Disc angle extension	12.0 (4.4)	10.4 (5.0)	0.114
Wedge angle	4.6 (5.2)	3.5 (4.9)	0.291
Lateral slippage	1.7 (2.6)	1.5 (2.5)	0.732
Range of motion	5.6 (3.2)	4.9 (3.7)	0.544
Follow-up			
Disc height	6.7 (2.8)	6.6 (2.5)	0.778
Vertebral body slippage	0.1 (4.8)	-0.3 (3.5)	0.628
Segmental lordosis	19.0 (11.6)	15.6 (10.1)	0.137
Disc angle flexion	7.7 (4.8)	5.1 (4.2)	0.006
Disc angle extension	12.2 (5.1)	9.7 (5.1)	0.024
Wedge angle	4.7 (4.7)	4.4 (5.7)	0.832
Lateral slippage	2.0 (2.4)	2.3 (3.4)	0.683
Range of motion	4.9 (3.7)	4.7 (3.4)	0.801
Change during follow-up			
D in disc height	-1.9 (2.4)	-1.5 (1.9)	0.280
D in vertebral body slippage	0.5 (4.3)	-0.5 (3.2)	0.216
D in segmental lordosis	0.3 (7.4)	0.4 (5.6)	0.901
D in disc angle flexion	1.2 (4.9)	-0.2 (3.4)	0.081
D in disc angle extension	0.2 (2.9)	-0.6 (3.3)	0.238
D in wedge angle	0.1 (4.9)	1.0 (3.5)	0.285
D in lateral slippage	0.3 (3.2)	0.8 (3.5)	0.085
D in range of motion	-0.6 (4.8)	-0.4 (3.3)	0.753

Radiographically we found significantly higher mobility of the segment above the fusion in extension, D: 12.2 (±5.1) vs. I: 9.7 (±5.1), p = 0.024, and flexion radiographs, D: 7.7 (±4.8) vs. I: 5.1 (±4.2), p = 0.006, of PLC-D patients, compared to those of the PLC-I group. All average values regarding preoperative measurements and final follow-up, as well as the average changes over time, are represented in Table 3.

**Table 3:** Levels fused PLC: posterior ligamentous complex

Levels fused	PLC disrupted, n (%)	PLC intact, n (%)
L1-L3	0 (0.00)	1 (1.06)
L1-L4	1 (3.57)	1 (1.06)
L1-pelvis	0 (0.00)	1 (1.06)
L2-L4	0 (0.00)	6 (6.38)
L2-L5	1 (3.57)	1 (1.06)
L2-S1	0 (0.00)	1 (1.06)
L3-L4	0 (0.00)	5 (5.32)
L3-L5	4 (14.27)	7 (7.45)
L3-S1	2 (7.14)	5 (5.32)
L3-pelvis	0 (0.00)	5 (5.32)
L4-L5	10 (35.7)	36 (38.3)
L4-S1	0 (0.00)	8 (8.51)
L4-S2	0 (0.00)	6 (6.38)
L4-pelvis	2 (7.14)	0 (0.00)
L5-S1	8 (28.57)	11 (11.7)
Total	28 (100.00)	94 (100.00)

## Discussion

Prevention of sASD following PLDF remains a challenge. Of the undoubtedly multifactorial nature of sASD, the specific role of the posterior ligamentous complex has been acknowledged in terms of its biomechanically stabilizing effect on the lumbar spine by a number of authors [3-5]. While ASD is usually treated nonsurgically, surgical treatment remains an option [6,9-12].

The PLC itself consists of multiple structures, such as the supraspinous and interspinous ligaments, facet joint capsules, and the ligamentum flavum [13]. Several clinical and biomechanical studies outline the structural importance of the PLC, particularly in the flexion of the lumbar spine [7,13-16]. Purposeful resection of the PLC may be needed in order to achieve neural decompression at the rostral end of the construct or even higher by performing a laminectomy above the fusion. However, the PLC at the rostral segment may be disrupted during routine surgical techniques by removing the entire spinous process of the UIV despite the absence of a specific indication. Preserving this natural soft tissue tension band between the UIV and UIV+1 may help provide additional physiologic stability and reduce the development of ASD and sASD by providing "minimal tissue disruption" in an elective lumbar surgical fusion setting [17].

In a cadaver study, increased upper adjacent level instability in conjunction with greater intervertebral disc stress was found after disruption of the PLC above a PLDF [18]. Our results underscored these findings in this focused retrospective clinical and radiographic study. Despite our relatively small cohort, our data demonstrated a radiographic increase in mobility of the segment above the fusion in dynamic radiographs of patients with an iatrogenically PLC-D. Moreover, we found that patients with a PLC-D between the UIV and UIV+1 had higher revision rates due to sASD than patients with a PLC-I during our observation period. The surgical revision rate of PLC-D patients exceeded the revision rate of PLC-I by 4.3%. This was, however, not a statistically significant finding, most likely due to the small sample size. Our radiographic indicators of ASD (including increase in intervertebral body slippage, decrease in DH ≥20%, decrease in ROM ≥20%, increase in lateral slippage, and increase in wedge angle) all showed higher incidences in patients with PLC-D, compared to patients of PLC-I group in Table 1.

Therefore, the findings of our study fully support current studies that suggest PLC-I is structurally relevant despite showing no significant differences in radiological findings between groups of PLC-I and PLC-D patients.

Heo et al. reported a revision rate of 8.7% with sASD in 378 patients who received L4-L5 or L5-S1 fusions due to spondylolisthesis. They identified several risk factors for the development of ASD. These include older age at the time of surgery, lower lumbar lordosis, lower segmental lordosis of the fused segment, preexisting disc degeneration of the UIV+1-segment, and preexisting facet-joint degeneration [19]. The revision rate reported by Heo et al. supports the overall revision rate of 7.4% due to sASD that we found in this study within the first 2.4 years (±1.7 years) after initial surgery [19].

Sun et al. performed a retrospective study undergoing posterior fusion due to spondylolisthesis. Forty-two patients were separated into two groups: one group represented a PLC-D in combination with a multisegmented decompression and fusion (multilevel group: 22 patients), while the PLC of the patients of the other group remained intact, and patients received only a single-segment decompression (single segment group: 20 patients). The authors reported ASD in only three patients of the multilevel group, without statistical significance, within three years of follow-up [17]. Sun et al. also reported a revision rate due to sASD of 7.1% (three of 42), consistent with the findings of Heo et al. (8.7%) and our own data (7.4%) [17,19]. The study presented by Sun et al. found all patients who required a revision due to sASD were part of the deficient PLC group [17]. However, our data showed that patients of group I (PLC-I) were also among the revision cases. This difference in the incidence of ASD could be attributed to the difference in the sample size of both studies (42 Sun et al. vs. 122 patients analyzed in our study), with a higher incidence observed in the PLC-D cohort shown in Table 1.

A retrospective chart review by Aono et al. examined 71 patients after PLIF of L3/4 with a long follow-up period (mean 5.8 years) [20]. The study showed a radiological incidence of ASD of 48%, sASD of 15%, and 8% of surgically treated ASD [20]. The indicators for ASD in our study denote higher incidences of ASD than reported using the criteria applied by Aono et al. [20]. However, we did not have a separate sASD group managed without surgical revision. Our actual revision rate for sASD in our study was remarkably similar to that reported by Aono et al. (8% and 7.4%) [20].

A retrospective study by Li et al. on 89 patients who received either decompression through bilateral laminectomy with disruption of the PLC or hemilaminotomies with preserved PLC integrity at the UIV (comparable to the midline-sparing laminotomy-subgroup in this study, Figure 1) found a significant difference in the incidence of revision surgery due to sASD at the UIV+1 [4]. Their data showed that 9% of the patients with a PLC-I compared to 36% of the patients with a deficient PLC returned to surgery due to sASD (study period 12.6 years) [4]. Unfortunately, their article was published in Chinese language, so no further comparisons could have been made with this very similar study. Based on their statistically significant results, the authors concluded that PLDFs with preservation of the PLC at the UIV+1 had lower rates of revision surgery for ASD compared to PLDFs where the PLC had been sacrificed [4]. Our results are consistent with these findings.

In the past two decades, there have also been other approaches to better predict the development of ASD. For example, Hikata et al. investigated whether the development of sASD at the UIV+1 level could be counteracted by a prophylactic midline sparing decompression in a retrospective study of 54 patients who underwent PLIF of L4/5 [21]. In 37 patients with mild stenosis of the UIV+1, concomitant decompression of the UIV+1 had been performed prophylactically [21]. In doing so, the authors demonstrated that preemptive decompression of the UIV+1 would reduce the incidence of sASD at the rostral segment of the fusion. They found that 57% of the patients showed radiological criteria for ASD at follow-up. Sixteen percent of the patients decompressed at the UIV+1, compared to 6% of the patients without any decompression above the fusion, developed sASD [21]. Hikata et al. concluded that a prophylactic decompression at the UIV+1 did not reduce the incidence of sASD [21]. The study, however, does not reveal how the PLC was managed between UIV and UIV+1, both in the decompression group and in the PLIF-only group. Conversely, one may conclude that surgical disruption of the posterior midline structures in the area of ​​the dorsal column at the UIV and UIV+1 seemed to be associated with an increased incidence of sASD. This conclusion is supported by the results obtained from the cadaver study mentioned above [18].

Liu et al. prospectively examined 120 patients who were planned for PLIF and randomly assigned them into three groups [7]. The group with bilateral decompression and resection of the spinous process showed signs of ASD in 71% and a revision rate due to sASD of 41%, compared to 12% ASD and 0% revision rate in the group that received an undercutting decompression after facet joint resection with preservation of the PLC. The authors found these results to be significant [7].

In our study, we focused specifically on the role of PLC integrity on spinal mechanics as seen on radiographs and surgical revision rates without addressing possible confounding variables. Although our study supports the current literature regarding the role of PLC in the development of ASD, there are some limitations to consider. The development of ASD is a multifactorial complex disease process of the spine with many risk factors, including the preservation of the PLC. Our retrospective study includes limitations such as potential selection bias and systematic errors inherent to the nature of the study. Furthermore, our study had unbalanced cohort sizes and a small-sample size with limited follow-up period due a loss of follow-up. This may explain the lack of statistical significance regarding PLC integrity at UIV+1 and the development of ASD and sASD. Further, we did not systematically collect patient-reported outcomes during the follow-up period. This led to the adoption of a general definition for sASD, with additional revision surgery necessary during the evaluation period of this study as inclusion criteria. Our small study population, although favorably sized in comparison to other studies on this subject, limits its generalizability. Overall, a larger study cohort with longer term observation windows would be necessary to try to answer the role of PLC preservation adjacent to an elective posterior lumbar fusion. Future research may explore additional spinal parameters and risk factors.

## Conclusions

Our retrospective clinical study showed an increase in the mobility of the segment above a lumbar fusion construct and a higher rate of revision surgeries above the fused levels in patients with PLC-D structure between the UIV and above. Within our observation period, we found that iatrogenic sacrifice of the PLC contributed to the development of ASD through instability and could in itself be a major contributor to higher surgical revision rates for ASD.
